# Comment on Budryn et al. Hydroxybenzoic Acids as Acetylcholinesterase Inhibitors: Calorimetric and Docking Simulation Studies. *Nutrients* 2022, *14*, 2476

**DOI:** 10.3390/nu14224859

**Published:** 2022-11-17

**Authors:** Ilham Zarguan, Abdelaziz Benjouad, Lamiae Belayachi

**Affiliations:** International Faculty of Medicine, College of Health Sciences, International University of Rabat, Rabat 10000, Morocco

It is with great interest that we read the article entitled “Hydroxybenzoic Acids as Acetylcholinesterase Inhibitors: Calorimetric and Docking Simulation Studies” published in *Nutrients* 2022, *14*, 2476 [1]. We found the article to be of utmost relevance considering the fact that the current anti-AD therapeutic arsenal approved by the FDA consists of only four drugs: memantine, rivastigmine, donepezil, and galantamine. However, these inhibitors have various side effects. Therefore, there is a serious need to develop an effective carrier system for the delivery of drugs to combat such a disease.

The study’s goal was to demonstrate the theory that hydroxybenzoic acids had strong reversible cholinergic inhibitory action. This may aid in the potential inclusion of functional products enriched with natural AChE inhibitors into the diet, which may be an effective approach in the symptomatic therapy of dementia. The authors evaluated the activity of 16 hydroxybenzoic acids in phytochemicals found in food based on epidemiological observations of plant products with anti-neurodegenerative effects.

The main issue we need to highlight and contend with is the study design. The baseline assays and strategies for finding AchE inhibitors must go through biochemical assays or computational studies (or combined) and then be evaluated in cell-based assays as well as in vivo assays. In the study by Budryn et al., ITC, a label-free method, was performed to determine the protein–ligand interactions and quantify the thermodynamic parameters. This method can also be used to characterize the nature of these interactions (e.g., binding affinity, entropic contributions, and enthalpic contributions). However, large amounts of protein are required for ITC, and it has many disadvantages. For this reason, combining ITC with Structure-Based Drug Design (SBDD) is generally the most useful method of elucidating the mechanism of inhibition, as it is time- and cost-effective. However, SBDD and ITC are both low-throughput methods. Therefore, the addition of a high-throughput screening method such as cholinesterase’s inhibition assay, enzyme selectivity and kinetic studies (as were performed in [2,3]), or fluorescence spectroscopic measurements [4] should be considered. Moreover, we recommend pre-calculating the pharmacokinetic properties including the absorption, distribution, metabolism, excretion, and toxicity (ADMET) of the selected compounds, as was addressed in a similar study [5]. We should also stress the importance of the reevaluation of Lipinski’s rule of five. 

In addition, the results were compared with known AD drugs based on previous studies in the literature, although it would have been more interesting to purchase one of these inhibitors as well and use it as a positive control under the same conditions as the tested compounds. The same applies to molecular docking, since it is necessary to compare the affinity obtained in the study with a known natural AChE inhibitor. 

We are looking forward to reading your results based on more cell-based assays using complex cell models. Further in vivo testing is also recommended to fully characterize the pharmacokinetics, bioavailability, optimal method of administration, and efficacy of these novel plant-based compounds.
